# The Utility of Multiple-Choice Assessment in Current Medical Education: A Critical Review

**DOI:** 10.7759/cureus.59778

**Published:** 2024-05-07

**Authors:** Priya Parekh, Vikesh Bahadoor

**Affiliations:** 1 Trauma and Orthopaedics, Wirral University Teaching Hospital, Wirral, GBR

**Keywords:** neurodiversity, test reliability, validity, utility, medical education, curriculum assessment, multiple-choice questions

## Abstract

In recent years, healthcare education providers have boasted about a conscious shift towards increasing clinical competence via assessment tests that promote more active learning. Despite this, multiple-choice questions remain amongst the most prevalent forms of assessment. Various literature justifies the use of multiple-choice testing by its high levels of validity and reliability. Education providers also benefit from requiring fewer resources and costs in the development of questions and easier adaptivity of questions to compensate for neurodiversity. However, when testing these (and other) variables via a structured approach in terms of their utility, it is elucidated that these advantages are largely dependent on the quality of the questions that are written, the level of clinical competence that is to be attained by learners and the impact of negating confounding variables such as differential attainment. Attempts at improving the utility of multiple-choice question testing in modern healthcare curricula are discussed in this review, as well as the impact of these modifications on performance.

## Introduction and background

Multiple-choice question (MCQ) tests are amongst the most commonly used method of assessment in healthcare education [1]. Traditional MCQs consist of two parts: a stem containing a problem or question and a set of options, one of which is correct. Other variations also exist, such as extended matching questions, true or false questions and script concordance tests [2].

With increased utilisation and development of practical assessment methods such as Objective Clinical Structured Examinations (OSCEs), direct observations of procedural skills and work-place based assessments, the use of MCQ testing is under greater scrutiny. The usefulness of MCQ testing can be critically analysed using the utility equation. This was first reported by van de Vleuten in 1996, who suggested that the utility of assessment methods can be evaluated by looking at the following five competing elements: validity, reliability, educational impact, costs and acceptability [3].

In this review, the elements of the utility equation are discussed for MCQ assessment in healthcare education, with a particular focus on validity and reliability. Beyond the utility equation, the impact of neurodiversity and use of feedback are also considered, as these topics have become increasingly significant for institutions in healthcare education in recent years [4,5].

## Review

The utility equation

Validity

The measure of validity in MCQ assessment in healthcare is multifaceted and depends on the type of validity being considered. Content validity refers to the extent to which the assessment method is representative of a curriculum [6]. MCQs are generally recognised as having a high content validity, and this is largely influenced by the common use of blueprinting in healthcare education [7,8]. Blueprinting usually involves the use of matrices that form a visual representation of the learning outcomes and the skill domains that are to be tested during assessment [9]. This is easy to refer to when formulating a question bank, as assessors can check off aspects of the blueprint. Content validity is increased by ensuring that maximal quantity of the blueprint is utilised and the spread of topics asked across the curriculum is equal [10]. Consequently, the use of blueprinting for MCQ assessment continues to be encouraged by various regulatory healthcare bodies, as it provides a more systematic and measurable approach to assessment which has high content validity. For example, the General Medical Council, which regulates and assesses all medical school curricula in the United Kingdom, specifies that all medical schools should have schemes of assessment mapped against desired outcomes across specialities or disciplines [11,12]. Despite this, it must be recognised that MCQs may lack content validity in healthcare education, where the development of other skills such as communication, collaborative working and practical skills are also essential components of curricula [11,13]. Miller’s pyramid of clinical competence is a schematic diagram describing a learner’s competence as a hierarchical process where, in increasing levels of competence, they are able to ‘know’ (knowledge), ‘know how’ (show competence), ‘show how’ (perform) and ‘do’ (action). MCQs are knowledge-based and are recognised in fulfilling lower levels (‘knows’ and ‘knows how’) of hierarchical learning models such as Miller’s pyramid [14]. Therefore, non-knowledge-based domains of healthcare curricula may be better tested by other forms of written assessment such as essays, or more practical methods of assessment to increase content validity, for example, by direct observation of skills [9].

The construct validity of MCQs, which refers to underlying unobservable characteristics or behaviour and how it aligns with the competency that requires assessment, is often scrutinised [13,15]. This is largely due to the impact of construct-irrelevant variance (CIV), defined as the erroneous inflation or deflation of correctly answered MCQ’s by uncontrolled variables. One example of CIV is a poorly written question that provides too much information and unintended cues in an item, which may erroneously inflate a correct answer. Another prevalent example is the impact of guessing, as all MCQ assessments involve a finite number of answers and therefore there is always some statistical probability (usually 20-25%) of guessing the correct answer. In both examples and others, various literature argues that the impact of CIV can and should be somewhat reduced by the test creator [15,16]. Techniques to develop more construct-valid MCQs have become well-known, such as including balanced and unbiased wording amongst the question stem and answers and considering the impact of random guessing when standard setting the pass mark. However, it is also recognised that MCQ creators must invest more time, effort and training for effective question development, which may be a barrier to negating CIV [15].

A high predictive validity of MCQ assessment would indicate that candidates’ result in one test positively and equally correlates to their outcomes in subsequent tests or with their clinical performance. MCQs have generally shown some degree of predictive validity in healthcare education in various studies; however, it is important to look at the significance of this validity and compare it to other forms of assessment [17,18]. A study by Wakeford et al. suggested that candidates who scored worse on MCQ post-graduate medical examinations (Membership of Royal College of General Practice [MRCGP] and Membership of the Royal College of Physicians [MRCP]) had subsequent significantly higher rates of reported Fitness to Practice (FTP) sanctions issued by the General Medical Council [19]. An explanation for this correlation is that knowing more provides protection against problems and sanctions. However, it may also be argued that demonstration of knowledge in MCQ assessment does not necessarily indicate its application clinically. Furthermore, the study also recognises that clinical assessment of these examinations (widely referred to as Practical Assessment of Clinical Examination Skills [PACES] and Clinical Skills Assessment [CSA]) had a significantly better prediction for FTP sanctions in comparison to MCQs. This may suggest that it is of greater benefit to utilise more practical forms of assessment, which can assess domains beyond knowledge, such as professionalism and ethics, if we want high predictive validity to assess clinical performance. The predictive validity of MCQs appears to be more significant for the prediction of future performance in MCQ assessments (which remains knowledge-based) rather than for prediction of skills-based, technical and non-technical assessments [19,20]. Significant predictive validity is commonly seen in undergraduate medical school education, where spaced-repetition MCQ tests are commonly utilised with an aim to demonstrate progress as the student learns more during each academic semester [21]. However, this assessment style may also be challenged as improved assessment scores may be influenced by memorisation of questions or question styles and cheating by formulation of question banks rather than increased acquisition of knowledge [20,22].

Reliability

The reliability of MCQ assessment refers to the consistent reproducibility of results. Reliability can be more formally assessed using the ‘true score theory’, which highlights that the variance in an individual’s score is due to differences in ability and errors in measurement:

X = T + E, Where X is the observed score, T is the true score and E is [random] error [23].

The true score is a theoretically perfect measure of a student’s ability, the random error is a measure of variables causing deviation from the true score and the observed score is the actual performance of a student [23]. MCQs are generally known to have high levels of reliability in comparison to other assessment methods when testing factual recall. This is suggested both anecdotally and statistically using measures of inter-rater, test-retest and internal consistency measures of reliability when compared to other forms of assessment in healthcare education [9,24-26]. However, several factors are also classically recognised to influence the ‘random error, ‘E’, of MCQs within the true score theory [23].

The reliability of MCQ assessment is reduced by differential attainment. Differential attainment describes the variation of results in assessment, training and recruitment due to factors outside of academic ability, such as gender, ethnicity, socio-economic status and age. Differential attainment has been increasingly recognised in undergraduate medical education, with some protected characteristics having more impact than others [27]. The largest study investigating longitudinal attainment gaps within written assessments (MCQs and short answer questions) during undergraduate medical education shows that attainment gaps significantly increased for international, non-white and male students, in comparison to other groups such as those with disability. Other studies show that this trend also continues during postgraduate medical education [27-29]. One explanation could be that disability is considered a ‘more-recognisable’ characteristic and there is greater encouragement to seek and provide support. If true, this also suggests that measures taken to protect individuals from differential attainment are impactful [29].

Factors leading to differential attainment in MCQ assessment may be explained by the deficit model, which suggests that underperformance is due to deficits in trainees. For example, international students may have additional language barriers to understand questions, feel less sense of belonging to study alongside peers or experience unconscious bias from teachers who have lower expectations of their score [28]. As compelling evidence becomes more available to highlight attainment gaps, increased measures have been implemented by regulatory bodies and educators [27]. For example, Intercollegiate Committee on Basic Surgical Examinations (ICBSE) have undertaken a linguistic review of the MRCS part A question bank to identify and remove any language that might have a cultural bias [30]. Despite this, information on the impact of negating differential attainment is currently minimal and hopefully more literature will become available as measures are implemented, with the subsequent reliability scores of MCQ questions between cohorts [27].

Other influences on random error, E, can be elucidated by referring to the type of reliability [23]. Inter-rater reliability measures the consistency of student outcomes when tested by different examiners and may reduce in MCQs as a result of varying experience of assessment creators in knowledge and question-writing. Test-retest refers to the reproducibility of consistent MCQ results over time [31]. MCQs have higher test-retest reliability compared to other methods of assessment such as OSCEs, supervised learning events or essays as the questioning environment is controlled and the options that the candidates can choose are discrete [32]. Nevertheless, test-retest reliability can be reduced by having short durations between MCQ tests, as participants may recall information from the first test or too long, as participants could have changed in some way (for example, having life-stressors or change in motivation), which could also bias results [31]. Internal consistency reliability can be maintained by ensuring consistent difficulty levels between MCQ items comprising the assessment and, again, is generally higher than other forms of assessment [9]. One statistical method of internal consistency reliability is Cronbach’s alpha, where a coefficient of >0.70 is generally recognised as an ‘acceptable’ internal consistency of a high-stake examination, and although exceptions exist, most studies show that well-written MCQ assessments have an internal consistency of >0.70 [26,33]. Within healthcare education, internal consistency may be affected during times of curriculum changes or when individuals have varying exposure to certain topics due to varied clinical placements and teachers [34]. Methods to improve reliability of MCQs in healthcare education include increasing examiner training, increasing the number of test questions and increasing similarity of questions within a test. However, these suggestions do not come without extra time, resources and expenditure into question-making, and the latter comes at the expense of reducing test validity, which must be balanced by question-makers and recruiters [9,35].

Educational Impact, Costs and Acceptability

Assessment drives learning, but we must consider the desired level of learning in Miller’s pyramid when choosing assessment methods [3,14]. Poorly constructed MCQs can have low educational impact as candidates may guess answers from answer cues or develop pattern recognition skills between questions instead of learning content. More conscientious wording can negate these influences, but, technically, still only allow for lower level thinking skills [24]. This is in contrast to other methods of assessments that are higher on Miller’s pyramid, such as essays and simulation, where learner preparation would require deeper learning of individual topics via application of knowledge, which would ultimately also be required during real clinical practice [36]. Nevertheless, MCQs enable a large range of topics to be assessed, which may be more useful when the breadth of knowledge of large curricula requires demonstration, and this may justify its continually prevalent use in healthcare education [10].

The cost of well-designed MCQ development is initially higher as it involves time and training for the production of questions and high-quality distractors from writers. However, once question banks are developed, this cost dramatically reduces as further costs only involve time to ensure that questions remain consistent with guidelines and costs to print or deliver questions on relevant technological software [37]. It may be plausible to reduce the costs of MCQ development by including fewer high-quality distractors. MCQs usually have four to five options in undergraduate and postgraduate medical examinations, but studies show that there is actually no optimal number of distractors [38,39]. For example, one study testing 132 medical students (categorised into three groups based on their previous MCQ achievement levels) found that there was no significant difference in the validity of student scores between three-, four- and five-choice questions [39]. However, this study did have some limitations due to its small sample size, and the scores between groups (separated by their achievement levels) were statistically different, which poses the question of how this concept would apply on a large scale of MCQ testing for a population with even greater variance in pre-existing achievements [37].

The acceptability of an assessment method refers to the wider justification of its use [3]. The high validity, reliability and cost-effectiveness of MCQ testing as described can be justified to all stakeholders creating assessments [40]. However, comparison of MCQ assessment becomes less acceptable with the formation of newer assessment methods that encourage higher order thinking skills such as high-fidelity simulation and virtual reality teaching. Nevertheless, MCQs have endured as a major component of healthcare education, probably due to its efficiency in knowledge acquisition compared to these other methods, as it cannot be disputed that a vast amount of knowledge underpins the competencies of a healthcare professional [3,37].

Neurodiversity

From 2002 to 2018, approximately 4.6% of medical students and newly qualified junior doctors in the United Kingdom declared a specific learning disability such as dyslexia, dyspraxia and attention-deficit/hyperactivity disorder [5]. Legislation in the United Kingdom requires higher education institutions to be anticipatory of the needs of students with disabilities, and this includes making reasonable adjustments during MCQ assessment. Reasonable adjustments include providing extra time, larger texts and computer-based software instead of hand-written answer sheets, and with the increasing prevalence of declared disability, it remains important to ensure that these measures allow students to perform up to their capabilities [1].

A study by Ricketts et al. in 2010 showed that there was no significant difference between mean MCQ test scores of more than 900 undergraduate medical students with learning difficulties and reasonable adjustments provided versus students with no learning difficulty. This study represented a large cohort and more than 1,000 MCQs comprising eight tests [1]. However, it only focussed on one specific medical school with specific reasonable adjustments, and these adjustments are not standardised with other higher education assessment providers in healthcare. In addition, students who had declared specific physical disabilities such as epilepsy, asthma and diabetes (which may contribute to neurodiversity) were excluded from the study. Nevertheless, the study does show that reasonable adjustments for MCQ assessment can provide equity in student performance outcomes. The key may be to ensure that optimal adjustments are established within each institution and for each specific MCQ test, but this provides a higher workload for test regulators and the impact of adjustments may not be apparent for each cohort until after the assessment and adjustments have been provided [41].

Feedback from MCQs

Reflection of feedback can drive further learning, and therefore it is important to provide feedback in the form that will fuel the most valuable reflection [42]. Feedback from MCQ assessment can be provided in several ways: pass or fail scoring, a quantitative score of an overall assessment as a raw mark or percentage, a quantitative score of individual topics or themes within the assessment or individualised explanations for each question. It is widely agreed that more detailed feedback beyond a quantitative score is more useful to the learner [43]. However, a study by Ryan et al. in 2020 showed that there is no significant benefit in providing conceptually focussed feedback (giving detailed discussion of the correct response) over response-oriented feedback (a brief explanation of why an answer is correct or incorrect) [4]. This highlights that more feedback does not always equate to better learning from MCQ assessment. One explanation for this is that succinct knowledge-based information is satisfactory to answer future MCQ questions, which remain knowledge-based [4]. This is in contrast to other forms of assessment, such as long-answer essays or practical simulation sessions, where conceptual feedback appears to produce better learner outcomes [44,45].

## Conclusions

When considering all elements of the utility equation, neurodiversity and feedback, the role of the question writer and healthcare institution appears significant in ensuring the optimisation of each factor. This is recognised by regulatory bodies in healthcare, such as the General Medical Council, which encourage the use of blueprinting to increase validity and undergraduate departments, which provide reasonable adjustments to reduce the effect of aspects of differential attainment in MCQ testing. Despite this, there remains further need to improve MCQ assessments, as factors such as reliability are continually compromised, for example, by ethnicity and sex, leading to differential attainment.

MCQs remain the most common method of assessment in undergraduate medical education and is a major component of postgraduate examination. Compared to other methods of assessment, the utility of MCQ testing can be justified by its high validity and reliability, but this is only applicable for certain aspects of curricula which are more knowledge-based and when question writing is of high quality to avoid any biases. The shortfalls of MCQ testing are repeatedly apparent when higher-level learning skills from Miller’s pyramid are desired. These shortfalls can be addressed by supplementing MCQs with other forms of assessment such as OSCEs, supervised learning events and simulation assessment, and this factor should be recognised by providers of healthcare education and beyond.
